# Analysis of metabolites change from reflorescence buds of ‘Cuiguan’ pear (*Pyrus pyrifolia*) based on LC-MS/MS

**DOI:** 10.3389/fpls.2025.1624304

**Published:** 2025-11-21

**Authors:** Bifeng Zhong, Linjia Jiang, Yun Tian, Wengui Li, Min Zhong, Quanjun Zhang

**Affiliations:** Institute of Horticulture, Sichuan Academy of Agricultural Sciences, Chengdu, China

**Keywords:** pears (*Pyrus pyrifolia*), reflorescence, defoliation, metabolomics, buds

## Abstract

Developed buds of pears (*Pyrus pyrifolia*) remain in a dormant state and do not break dormancy until the following spring. However, climatic factors and leaf diseases trigger defoliation in late summer and early autumn, which typically results in the bud paradormancy release and subsequent sprouting. This not only depletes tree nutrients but also diminishes the quantity of flowers and the yield of fruit in the following summer. In this study, metabolic changes in ‘Cuiguan’ pear buds were investigated following premature leaf fall. A total of 1,533 metabolites were annotated, with the majority being downregulated. Sugar levels decreased during the release of paradormancy, likely to provide energy for subsequent growth. Concurrently, most amino acids were consumed post-defoliation, with only a few showing increased trends. Furthermore, the observed low levels of phenylpropanoid-related metabolites in flower buds may contribute to premature senescence. This metabolic profile contributes to our understanding of the biological mechanism of paradormancy release from defoliation, and clarifies the metabolic changes during this process.

## 1 Introduction

Pear, an important economical fruit within the family Rosaceae genus Pyrus, has thousands of cultivars worldwide (Wu et al., 2018). Pyrus pyrifolia, commonly known as the sand pear, is widely cultivated in southern China. With a cultivation area of approximately 8×10^4 hm² in Sichuan Province, pear accounts for over 17% of the province’s total fruit area, it produces an annual output of about 9×10^5 t (Zhang et al., 2023). In China, pear buds typically break dormancy and bloom from March to May, set fruit in autumn, and then enter a dormancy state to overwinter. Bud dormancy is categorized into three types: ecodormancy, endodormancy, and paradormancy (Lang et al., 1987). By the middle and late June, buds complete physiological differentiation (Tromp, 2000), after that floral organ primordia gradually develop, reaching full maturity by autumn. Subsequently, buds enter a brief paradormancy period, transitioning into endodormancy as temperatures drop and leaves senesce, ultimately entering ecodormancy to overwinter.

In recent years, sand pear cultivars such as ‘Cuiguan’ have exhibited abnormal physiological phenomena, including premature leaf senescence and early defoliation. These conditions lead to untimely bud release and subsequent blooming in autumn, a phenomenon known as reflorescence. In some cases, these autumn blossoms develop into weak fruits that persist into early winter but eventually fall as temperatures decline. According to field investigations, this reflorescence phenomenon results in significant yield reductions of 20-30% in the following year due to diminished bud reserves. This issue has become prevalent in Sichuan Province and across southern China. Similar observations have been reported globally, including studies from South Africa (Pierre et al., 2002), New Zealand (Sung-Do and David, 2003) and Japan (Tominaga et al., 2022), indicating that this is a widespread concern in pear cultivation worldwide.

Previous researches on reflorescence have primarily concentrated on elucidating its underlying causes and exploring potential solutions. Extensive study has demonstrated premature defoliation as the primary trigger, influenced by a complex interplay of endogenous and exogenous factors. From an endogenous perspective, suboptimal nutritional status, excessive fruit load, and improper pruning practices have been demonstrated to induce autumnal defoliation (Ruan, 2014). Additionally, genetic predisposition plays a crucial role, with certain cultivars such as ‘Cuiguan’ and ‘Fengshui’ exhibiting heightened susceptibility to autumnal defoliation, while others like ‘Huanghua’ and ‘Huanong 1’ demonstrate greater resistance to autumn reflorescence (Xiaoping et al., 2018). The exogenous factors contributing to defoliation primarily include foliar pathogens and adverse environmental conditions, particularly elevated temperature and humidity levels (Huang et al., 2015). Epidemiological studies have illustrated black spot (Alternaria alternata), anthracnose, and powdery mildew as predominant pathogens causing significant defoliation across Jiangsu, Anhui, and Shanxi provinces, with over 60% of leaves adversely affected (Yu, 2010).Regional studies in Zhejiang have revealed the prevalence of black spot, anthracnose, and rust on Pyrus pyrifolia leaves, which have induced defoliation early in the autumn (Yang et al., 2012). In Fujian province, comprehensive investigations have established a strong correlation between three major foliar diseases (pear spot disease, brown patch, and black spot) and premature leaf senescence, demonstrably showing positive correlations between disease indices and defoliation incidence across various cultivars, orchards, and temporal intervals (Huang et al., 2018). Jia has found that *Alternaria alternata*, the cause of black spot disease, is one of major factors for abnormal leaf senescence through ABA-mediated signaling pathways (Jia et al., 2021.). Collectively, these studies demonstrate that black spot, anthracnose, and rust are significant contributors to premature leaf aging and subsequent defoliation.

Current prevention strategies emphasize three main approaches: fungicide application, hormonal regulation, and cultivar selection. Fungicidal treatments effectively curb foliar infections, thereby prolonging leaf retention. Hormonal including NAA and ABA has effect after defoliation. Wei has improved that NAA application has been empirically demonstrated to reduce post-defoliation reflorescence rates (Wei et al., 2022). Exogenous ABA can compensate for the ABA loss caused by leaf removal. The GA3+IAA/ABA value decreases, thereby reducing flower bud budding and reflorescence (Zhang et al., 2016). Moreover, Strigolactones, owing to their bud growth-suppressing properties (Gomez-Roldan et al., 2008), have emerged as promising hormonal alternatives. However, these methods present significant limitations in terms of economic viability and environmental sustainability, underscoring the need for more ecologically sound and cost-effective solutions.

Maintaining pear buds in dormancy state during autumn is crucial for optimizing fruit yield. Given that premature defoliation triggers bud sprouting, it is evident that leaves play a pivotal role in sustaining bud dormancy. Wei demonstrated that bud sprouting following defoliation is predominantly influenced by the presence of leaves rather than other plant organs (Wei et al., 2022). This suggests that compounds or signals derived from leaves may serve as key regulators of bud dormancy. Consequently, the mechanisms underlying leaf-mediated regulation of bud sprouting induced by early defoliation necessitate further investigation.

In this study, we annotated differential metabolites associated with reflorescence, uncovering a total of 1,533 metabolites, comprising 898 in positive ion mode and 635 in negative ion mode. While previous metabolic analyses have highlighted the significance of sugars, amino acids, and secondary metabolites in dormancy release, our findings reveal distinct changes in these metabolite profiles under early defoliation conditions. Based on these observations, we propose a novel model for paradormancy release, which integrates the roles of sugar, amino acid, and secondary metabolite dynamics induced by early defoliation.

## 2 Materials and methods

### 2.1 Plant materials and treatments

The artificial defoliation treatment was performed using early-ripening pear cultivar “Cuiguan” *(Pyrus pyrifolia)* trees, which are prone to early defoliation. The trees were grown in Sichuan Academy of Agricultural Sciences demonstration orchard, Xindu district, Chengdu city, Sichuan province, China. Where the average temperature is 17.2°C, the average annual precipitation is 900 to 1,300 mm. Twelve healthy adult trees (6 years old) with no defoliation were selected for the artificial defoliation treatment, three of which were used for calculating bud break rates. The remaining 9 trees were evenly divided into 3 groups, with three trees serving as one biological replicate. All the leaves were manually removed on September 4, 2023. Ten spurs per tree were selected to determine the bud break rate. The state of sprouting was assessed according to the appearance of fresh bud tissues and the subsequent growth. Spur flower buds from treatment and control groups were sampled at 7, 14 and 21 days after defoliation, frozen in liquid nitrogen, and stored at –80°C until used. Treatment groups were marked as Cuiguan-sampling date, which has been abbreviated as CG-911; and control groups were marked as Mock-date, which has been abbreviated as CK-911.

### 2.2 Observation of tissue sections

Harvest pear flower buds from the leaf-removal treatment group and the control group. Fix them with 10% formalin-acetic acid-alcohol (FAA fixative) solution. Entrust Chengdu Lilai Biotechnology Co., Ltd. to carry out paraffin embedding and sectioning of the flower buds fixed with FAA. The sections are stained with safranin-fast green. Observe and take photos of the stained flower bud sections under a DM1000 LED microscope (Leica, Wetzlar, Germany).

### 2.3 Buds’ metabolite extractions and preparation

Those buds from the defoliated and control groups were used as experiment group and control group respectively. Each group had 6 biological replicates. Those buds were quickly frozen in liquid nitrogen immediately and grounded into fine powder with a mortar and pestle.1000 μL methanol/acetonitrile/H2O (2:2:1, v/v/v) were added to homogenized solution for metabolite extraction. The mixture was centrifuged for 20 min (14000g, 4°C). The supernatant was dried in a vacuum centrifuge. For LC-MS analysis, the samples were re-dissolved in 100 μL acetonitrile/water (1:1, v/v) solvent and centrifuged at 14000 g at 4°C for 15 min, then the supernatant was injected.

### 2.4 LC-MS/MS analysis

Metabolomic profiling was performed using an untargeted approach with liquid chromatography coupled with tandem mass spectrometry (LC-MS/MS). This strategy was adopted to provide a comprehensive, unbiased overview of the metabolic changes associated with reflorescence, allowing for the simultaneous detection of a wide range of metabolites without prior selection. This was particularly appropriate for our study as the metabolic basis of reflorescence is not fully elucidated, and our goal was to hypothesize novel metabolic pathways and identify potential biomarker metabolites. untargeted.

Analyses were performed using an UHPLC (1290 Infinity LC, Agilent Technologies) coupled to a quadrupole time-of-flight (AB Sciex TripleTOF 6600) in Shanghai Applied Protein Technology Co.,Ltd.

The samples were separated by Agilent 1290 infinity LC ultra performance liquid chromatography (UHPLC) on a C-18 column; The column temperature was 40°C. The flow rate was set at 0.4 ml/min; and the injection volume was 2 μL. The mobile phase A consisted of 25 mM ammonium acetate and 0.5% formic acid in water, mobile phase B was methanol. The gradient elution procedure was as follows: 0-0.5 min, 5% B; then B changed to 100% linearly from 0.5 to 10 min; 10-12. 0 min, B was maintained at 100%; From 12.0 to 12.1 min, B changed linearly from 100% to 5%; 12.1–16 min, B was maintained at 5%. During the whole analysis, the sample was placed in an automatic sampler at 4°C. In order to avoid the influence caused by the fluctuation of the instrument, the random sequence was used for the analysis of samples. Quality control (QC) samples are inserted into the sample queue to monitor and evaluate the stability and the reliability of the data.

The ESI source conditions were set as follows: Ion Source Gas1 (Gas1) as 60, Ion Source Gas2 (Gas2) as 60, curtain gas (CUR) as 30, source temperature: 600°C, IonSpray Voltage Floating (ISVF) ± 5500 V. In MS only acquisition, the instrument was set to acquire over the *m/z* range *60–1000 Da*, and the accumulation time for TOF MS scan was set at 0.20 s/spectra. In auto MS/MS acquisition, the instrument was set to acquire over the *m/z* range *25–1000 Da*, and the accumulation time for product ion scan was set at 0.05 s/spectra. The product ion scan is acquired using information dependent acquisition (IDA) with high sensitivity mode selected. The parameters were set as follows: the collision energy (CE) was fixed at 35 V with ± 15 eV; declustering potential (DP), 60 V (+) and −60 V (−); exclude isotopes within 4 Da, candidate ions to monitor per cycle: 10.

### 2.5 Data preprocessing

Raw MS data (.wiff.scan files) were converted to MzXML format using ProteoWizard MSConvert prior to processing with the open-source XCMS package. Peak detection was conducted using the centWave algorithm with the following parameters: *m/z* = 10 ppm, peakwidth = c(10, 60), and prefilter = c(10, 100). For peak grouping, the settings were bw = 5, mzwid = 0.025, and minfrac = 0.5. Isotope and adduct annotation were performed using the CAMERA (Collection of Algorithms of MEtabolite pRofile Annotation). Extracted ion features were filtered to retain only those variables with >50% non-zero measurements in at least one experimental group.

Putative metabolite annotation was carried out by querying experimental data against a plant metabolome database. Metabolites in the biological samples were structurally identified by matching their molecular mass (mass error < 10 ppm), MS/MS spectra, and retention time with those recorded in the database. As this study employed a non-targeted LC-MS/MS approach without the use of authentic chemical standards, all identifications are considered putative, corresponding to confidence Levels 2 and 3 according to metabolomics reporting standards.

### 2.6 Statistical analysis

After normalized to total peak intensity, the processed data were analyzed by R package (ropls, R version 3.6.3), where it was subjected to multivariate data analysis, including Pareto-scaled principal component analysis (PCA) and orthogonal partial least-squares discriminant analysis (OPLS-DA). The 7-fold cross-validation and response permutation testing were used to evaluate the robustness of the model. The variable importance in the projection (VIP) value of each variable in the OPLS-DA model was calculated to indicate its contribution to the classification. Metabolites with the VIP value >1 was further applied to Student’s t-test at univariate level to measure the significance of each metabolite, the p values less than 0.05 were considered as statistically significant. The KEGG analysis was performed for the significantly altered metabolites, KEGG Compound Database (http://www.kegg.jp/kegg/compound/) and KEGG Pathway Database (http://www.kegg.jp/kegg/pathway.html).

### 2.7 Metabolite annotation

This study utilized the plant metabolome database for metabolite annotation. The structural annotation of metabolites in the biological samples was performed by matching experimental data with database entries, based on key parameters including molecular mass (with a mass error tolerance of <10 ppm), secondary fragmentation spectra, and retention time. All annotations were subsequently verified and confirmed to ensure reliability.

## 3 Results

### 3.1 Defoliation advanced the development of spur flower bud to induce its break in autumn

**Figure 1:**
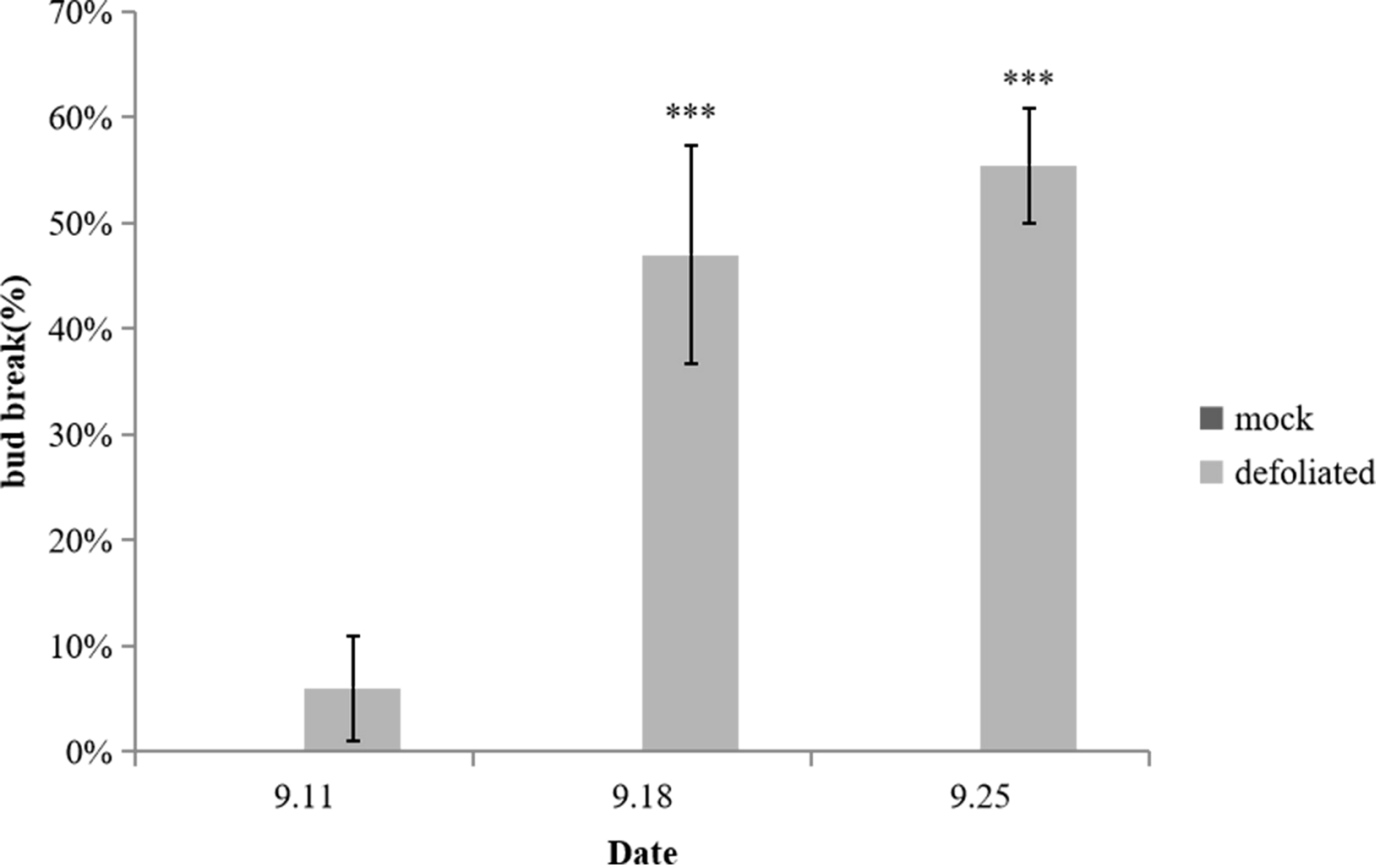
Bud break rate after defoliation. Error bars indicate the SEs of ten biological. Replicates and letter indicate significant differences between the mock control and defoliated trees (student’s test). ***P<0.001.

**Figure 2:**
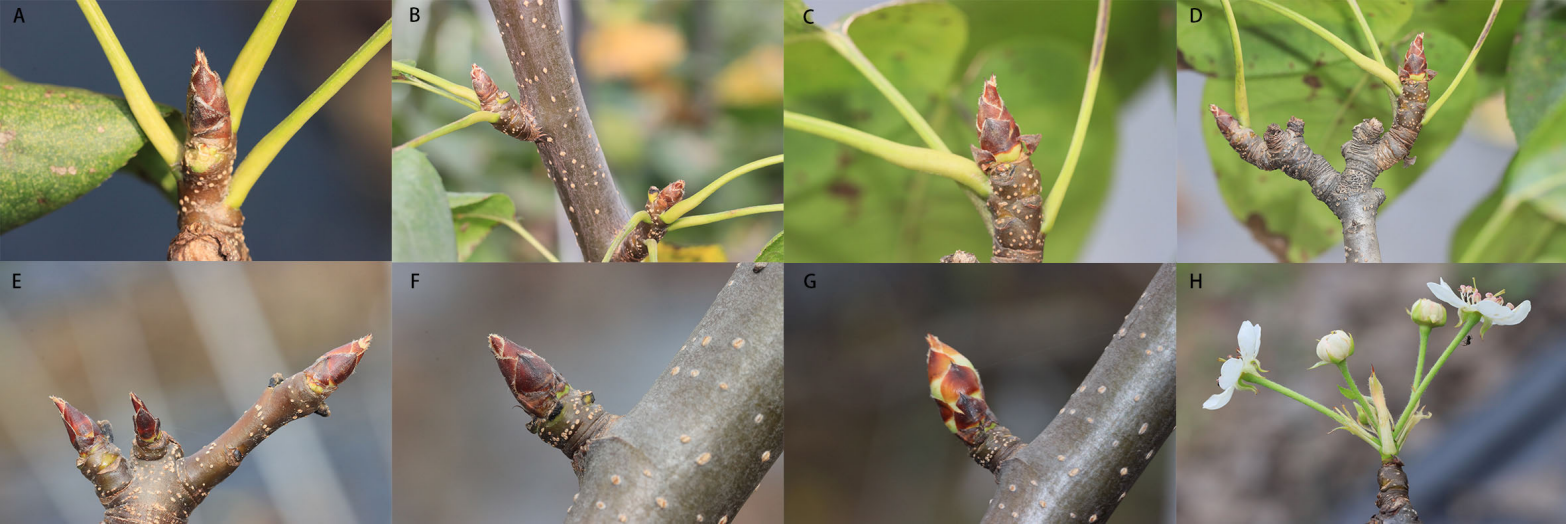
Early defoliation induced “Cuiguan” pear spur flower bud break in autumn. **(A-D)** The mock on 9.4, 9.11, 9.18 and 9.25 separated. **(E-H)** the defoliated trees’ buds on 9.4, 9.11, 9.18 and 9.25 separated.

To investigate the impact of defoliation on spur flower bud development, we removed all the leaves of the pear trees on 4th September (**Supplementary Figure 1**). Preliminary observations indicated that bud sprouting typically occurs within several days following early defoliation. To quantify this phenomenon, the bud break rate was systematically monitored over a 21-day period post-defoliation. Our experimental data demonstrated a marked increase in bud break rate following leaf removal. Notably, statistically significant differences (p < 0.05) in bud break rates were observed between the defoliation treatment and control groups at 7th, 14th, and 21th day post-treatment (**Figure 1**). The defoliation treatment significantly enhanced bud release, achieving bud break rate 6%, 47% and 56% during three weeks of observation. These findings provide compelling evidence that defoliation effectively induces spur flower bud break in pear trees (**Figure 2**).

**Figure 3:**
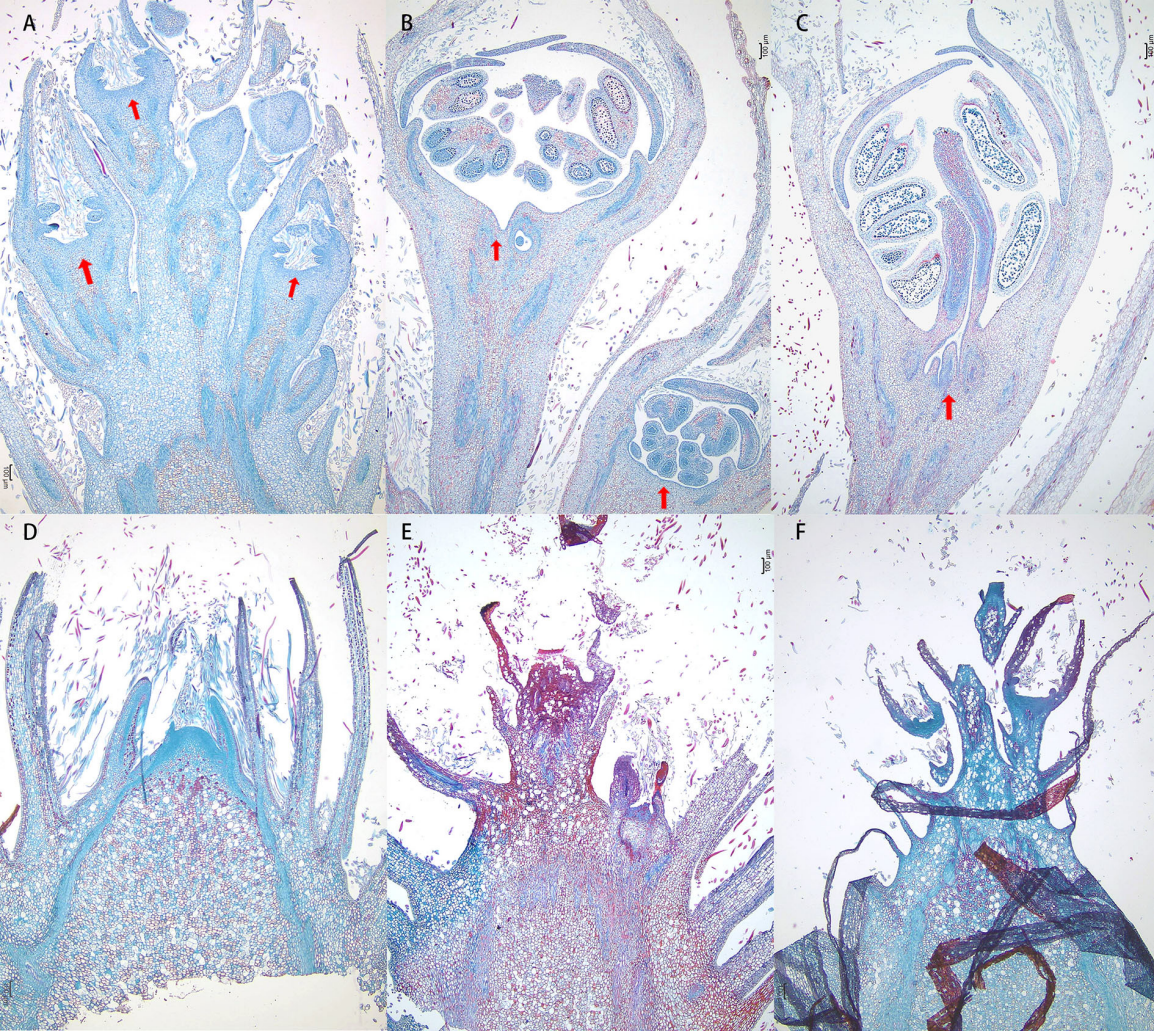
Defoliation accelerated flower bud development. Vertical sections of flower buds collected at 7 days **(A, D)**, 14 days **(B, E)** and 21 days **(C, F)** after defoliation from mock control trees and defoliated trees. Floral primordium are indicated by red arrowheads.

To investigate the impact of defoliation on the morphological development of flower buds, we conducted a detailed anatomical analysis. Our observations revealed that flower buds began to sprout as early as 7 days after defoliation, while no visible changes were detected in the control group during the same period. Upon examining the vertical sections of the flower buds, petal primordium was small and the pistil primordia cells were closely arranged at 7th day post-defoliation. At 14th day post-defoliation, the outline of butterfly anther becomes clear and the pollen grains were taking shape. At 21th day post-defoliation, the pollen grains were clearly visible (**Figure 3**). Moreover, we measured the area of the sprouted florets, which were 0.12 mm², 1.14 mm², and 1.75 mm², respectively. While the control buds were still in a state of pending development. These findings suggest that defoliation accelerates the developmental progression of flower buds with defoliation.

### 3.2 1,533 metabolites were annotated and quantitated by LC-MS/MS

#### 3.2.1 OPLS-DA model validation confirms high predictive accuracy and robustness

**Figure 4:**
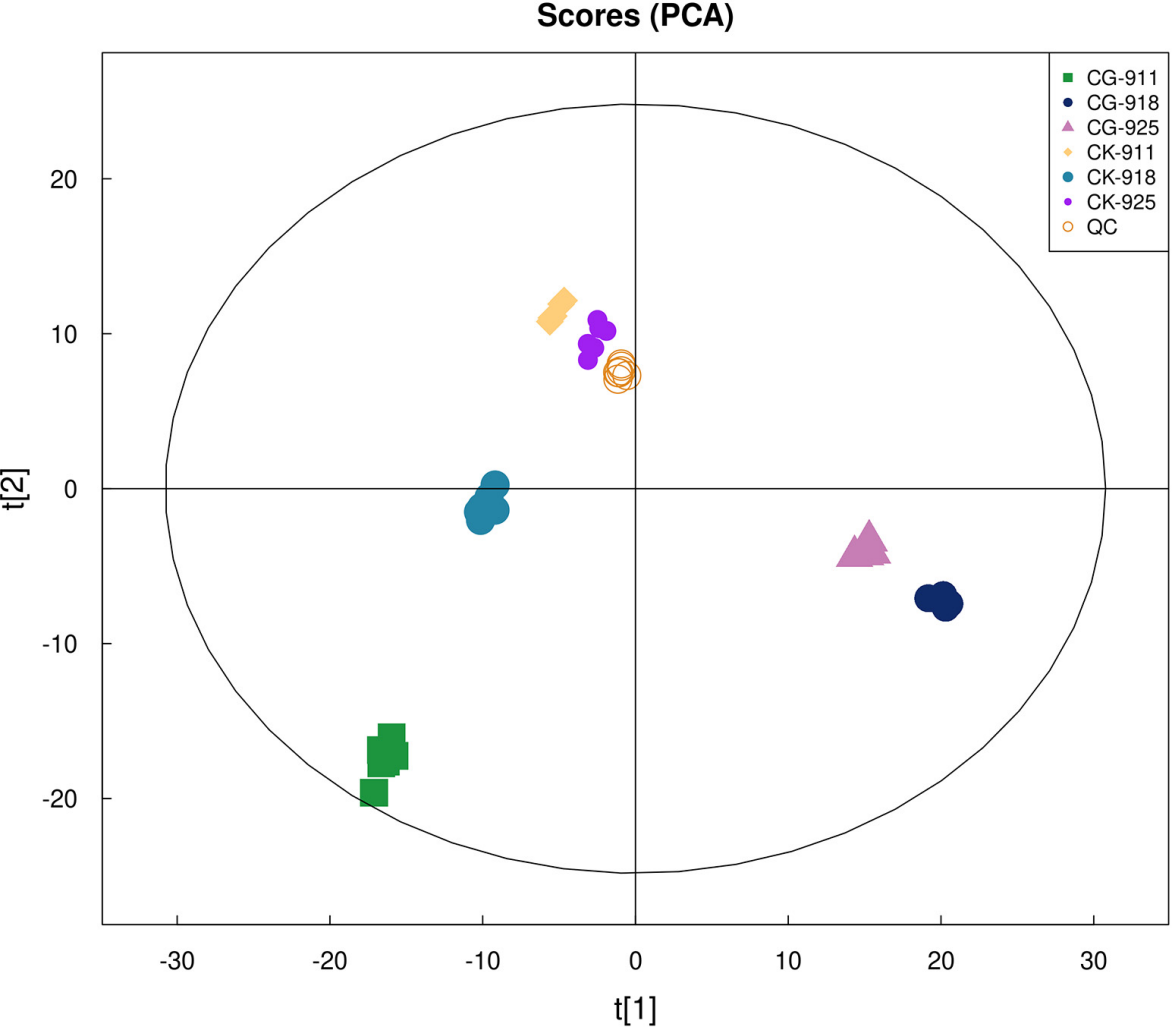
The PCA model score plots.

**Figure 5:**
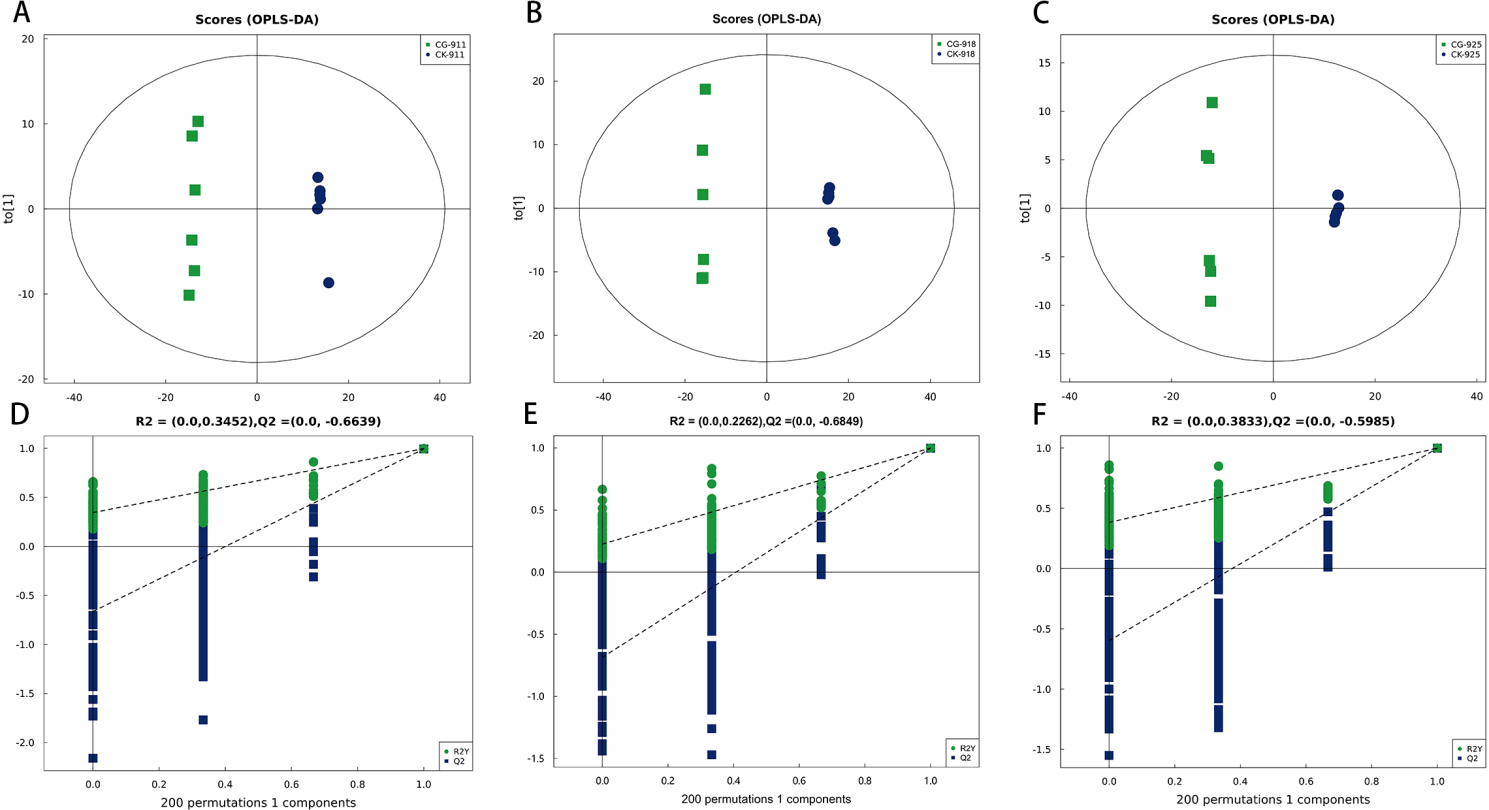
The OPLS-DA model score plots **(A-C)**, and permutation test of the OPLS-DA model **(D-F)** for comprehensive metabolomics data of pear buds. **(A, D)** CG-911 vs CK-911, **(B, E)** CG-918 vs CK-918, **(C, F)** CG-925 vs CK-925.

Principal Component Analysis (PCA) effectively illustrated the distinctions between the control group and the defoliation-treated pear buds. The PCA score plots demonstrated a clear separation between the defoliated groups and the control group (**Figure 4**). Buds samples with or without treatment from different time points were clearly separated, and tightly clustering of QC samples demonstrated high analytical reproducibility. To further enhance the differentiation between these groups, the Orthogonal Partial Least Squares-Discriminant Analysis (OPLS-DA) model was employed, with 7-fold cross-validation used to derive the model parameters (R²Y and Q²). All models yielded R²Y > 0.99 and Q² > 0.99 (**Figure 5**), indicating the strong explanatory power and predictive accuracy. To ensure the robustness of the OPLS-DA model and prevent overfitting, a permutation test was conducted. The permutation test results yielded intercepts of R² = 0.3452 and Q² = −0.6639 for group CG-911 vs CK-911, R² = 0.2733 and Q² = −0.5961 for group CG-918 vs CK-918, and R² = 0.3833 and Q² = −0.5985 for group CG-925 vs CK-925 (**Figure 5**). These findings confirmed that the OPLS-DA model exhibited high predictive accuracy without evidence of overfitting, validating its suitability for subsequent analytical investigations.

#### 3.2.2 Global view of metabolites changes with reflorescence

**Figure 6:**
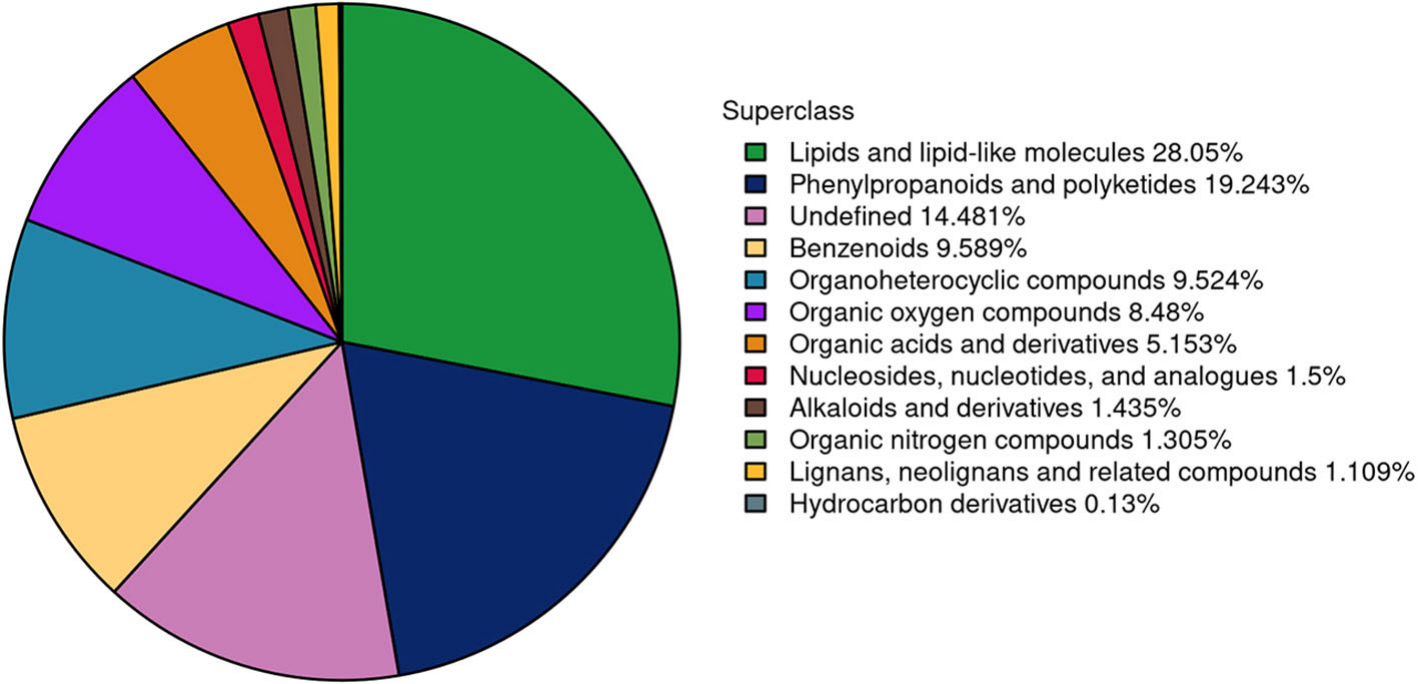
The number and proportion of metabolites annotated in each chemical classification.

The metabolic changes in pear buds during the transition from dormancy to bud release were systematically investigated. Using LC-MS/MS, the multi-peak profiles of metabolites across six groups of pear buds were analyzed. The chromatogram representing the metabolite profiles during the bud stage is presented in **Figure 6**. A total of 1,533 metabolites were annotated, comprising 898 in positive ion mode and 635 in negative ion mode, all of which may potentially play a role in bud release. These metabolites were classified into 12 categories: 22 alkaloids and derivatives, 147 benzenoids, 2 hydrocarbon derivatives, 17 lignans, neolignans, and related compounds, 430 lipids and lipid-like molecules, 23 nucleosides, nucleotides, and analogues, 79 organic acids and derivatives, 20 organic nitrogen compounds, 130 organic oxygen compounds, 146 organoheterocyclic compounds, 295 phenylpropanoids and polyketides, and 222 others.

#### 3.2.3 Altered metabolome in response to defoliation

##### 3.2.3.1 936, 1189 and 1223 metabolites changed during paradormancy release

To investigate the differential expression of metabolites between the control and experimental groups, we performed fold change analysis (FC > 1.5 or FC < 0.67) combined with t-test (p < 0.05) for the construction of volcano plots (**Supplementary Figure 2**). The analysis revealed significant alterations in bud metabolites across all experimental groups.

In the comparison between CG-911 and CK-911 groups, we found 224 up-regulated and 712 down-regulated metabolites. Among the up-regulated compounds, prenol lipids and organic oxygen compounds showed notable changes. Conversely, flavonoids, fatty acyls, and multiple organic oxygen compounds were predominantly down-regulated. In the CG-918 vs CK-918 comparison, we observed 293 up-regulated and 896 down-regulated metabolites. The up-regulated compounds included 51 fatty acyls, 23 carboxylic acids and derivatives, and 20 prenol lipids, with Pyrrole-2-carboxylic acid (FC = 103.01) and Ginkgolic Acid C15:1 (FC = 98.50) showing the most significant increases. In contrast, down-regulated metabolites comprised 135 prenol lipids, 84 flavonoids, 76 organic oxygen compounds, and 73 fatty acyls. For the CG-925 vs CK-925 analysis, 214 metabolites were up-regulated while 1009 were down-regulated. The up-regulated group included 14 fatty acyls, 7 organic oxygen compounds, and 5 carboxylic acids and derivatives. Among the down-regulated metabolites, we found 11 flavonoids, 10 prenol lipids, and 9 organic oxygen compounds.

The number of differential metabolites increased significantly during the flower bud releasing process, with most exhibiting markedly decreased expression, prenol lipids, flavonoids, fatty acyls, and multiple organic oxygen compounds showed the largest decreases and continued to reduce with reflorescence. This suggests that as pear buds develop, the later stages involve more complex metabolic pathways.

**Table 1** summarizes the metabolites with the highest VIP values (chosen top 3 highest values from each compare group) across the three time points. There are 5 metabolites have the highest VIP values, down-regulation: 9-octadecenamide, cianidanol, and octadecanamide, up-regulation: 4a-carboxylic acid and choline. From CG-918 vs CK-918 and CG-925 vs CK-925, the metabolites and their change model are exactly the same, so we speculated that 9-octadecenamide, choline, and octadecanamide have really important effect on reflorescence buds development.

**Table 1:** Differential metabolites with most VIP value after treatment.

Group	Name	VIP	Fold change	P-value
CG-911 vs CK-911	9-Octadecenamide	14.34	0.49	0.002
	4a-carboxylic acid	12.93	1.91	<0.001
	Cianidanol	11.65	0.60	<0.001
CG-918 vs CK-918	Choline	10.25	2.13	<0.001
	Octadecanamide	10.06	0.41	<0.001
	9-Octadecenamide	10.03	0.06	<0.001
CG-925 vs CK-925	Octadecanamide	10.66	0.20	<0.001
	9-Octadecenamide	9.91	0.45	<0.001
	Choline	9.90	1.34	<0.001

**Figure 7:**
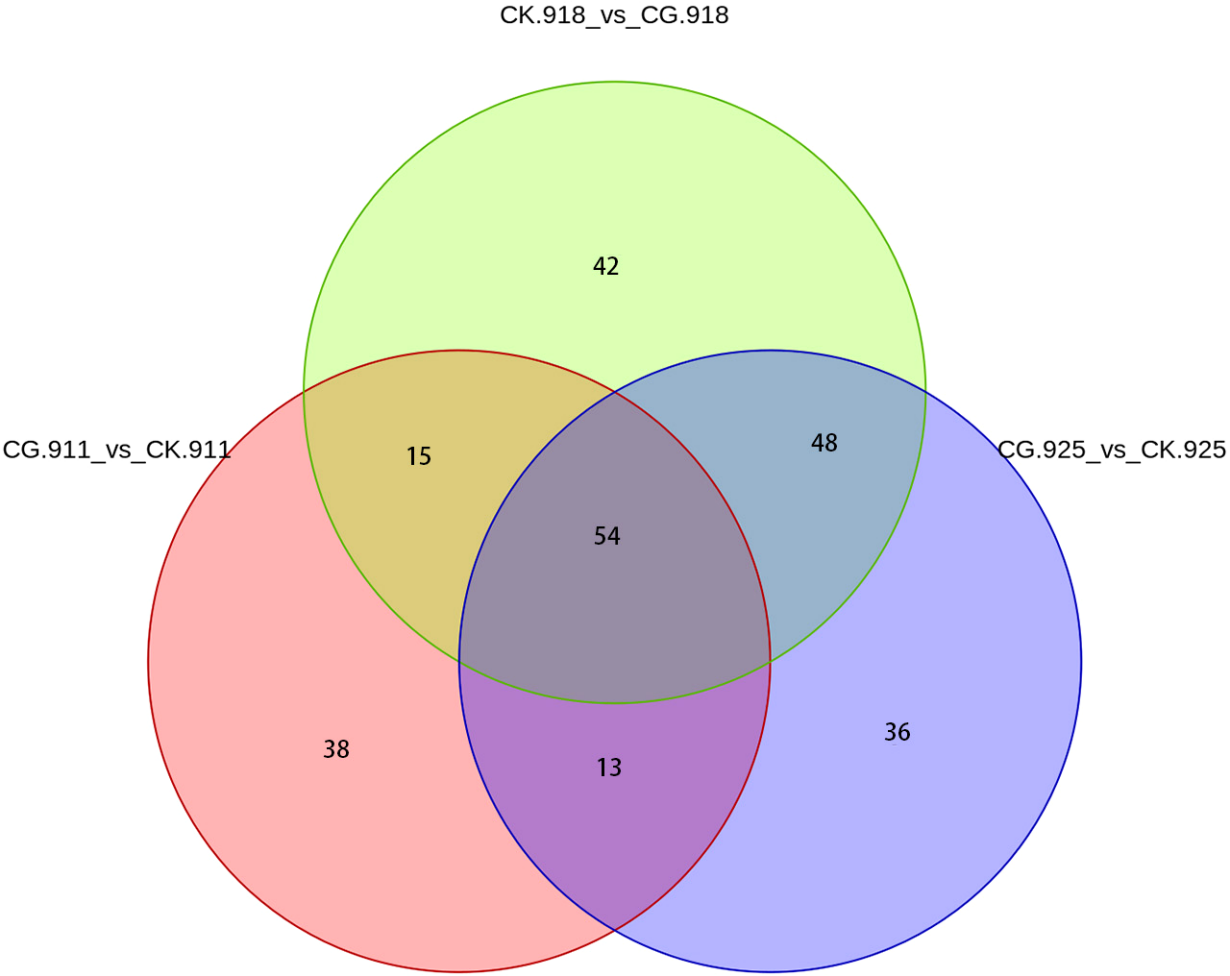
venn of 3 compared groups.

The Venn diagram analysis revealed that 54 metabolites were consistently present across all three comparison groups (**Figure 7**). These shared metabolites were categorized as follows: 3 benzenoids, 13 lipids and lipid-like molecules, 4 organic acids and derivatives, 2 organic nitrogen compounds, 9 organic oxygen compounds, 2 organoheterocyclic compounds, 11 phenylpropanoids and polyketides, and 10 others. This overlap highlights a core set of metabolites that may play crucial roles in the biological processes underlying the three experimental conditions.

##### 3.2.3.2 KEGG pathway Enrichment

Following the screening of significantly differential metabolites, the KEGG database (https://www.kegg.jp/) was utilized to analyze the metabolic pathways associated with these metabolites, aiming to identify perturbed biological pathways.

**Figure 8:**
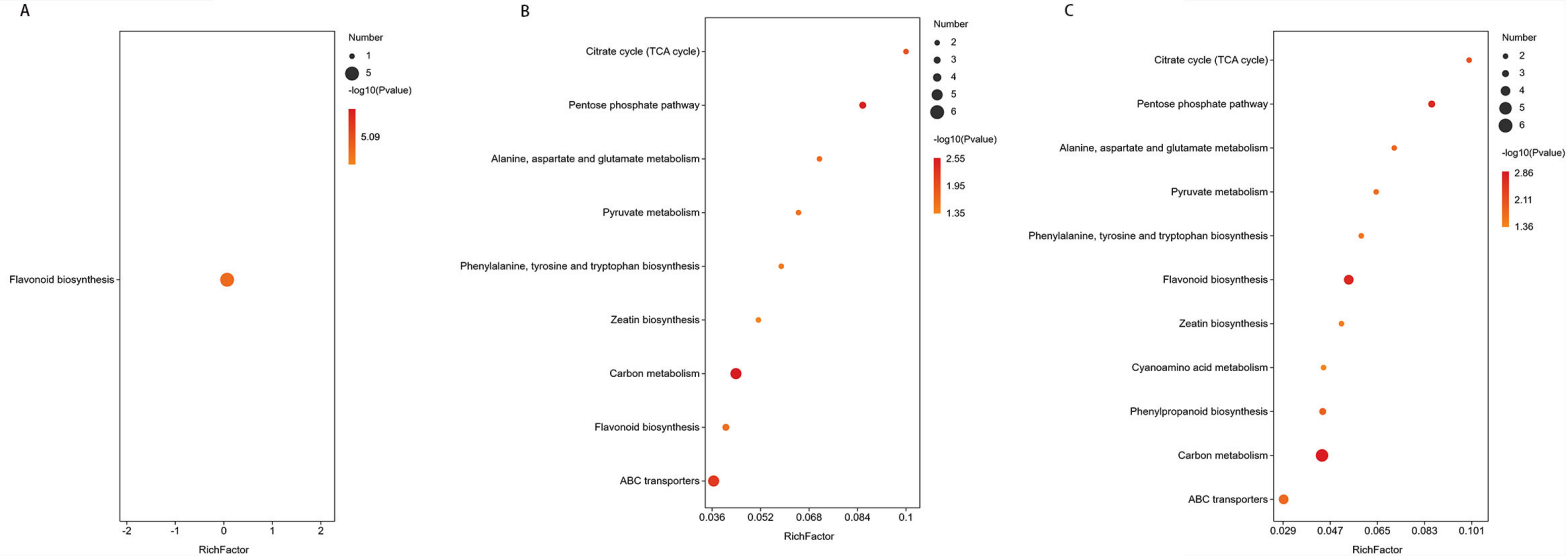
Metabolic pathway enrichment of differential metabolites. **(A-C)** represent the metabolic pathways enriched in the CG-911 vs CK-911, CG-918 vs CK-918 and CG-925 vs CK-925. The abscissa represents the Rich factor. The larger the graph, the more the number of different metabolites; the color indicates the significance of enrichment, that is, the p-value, the darker the red indicates that the pathway is more significantly enriched.

**Figure 9:**
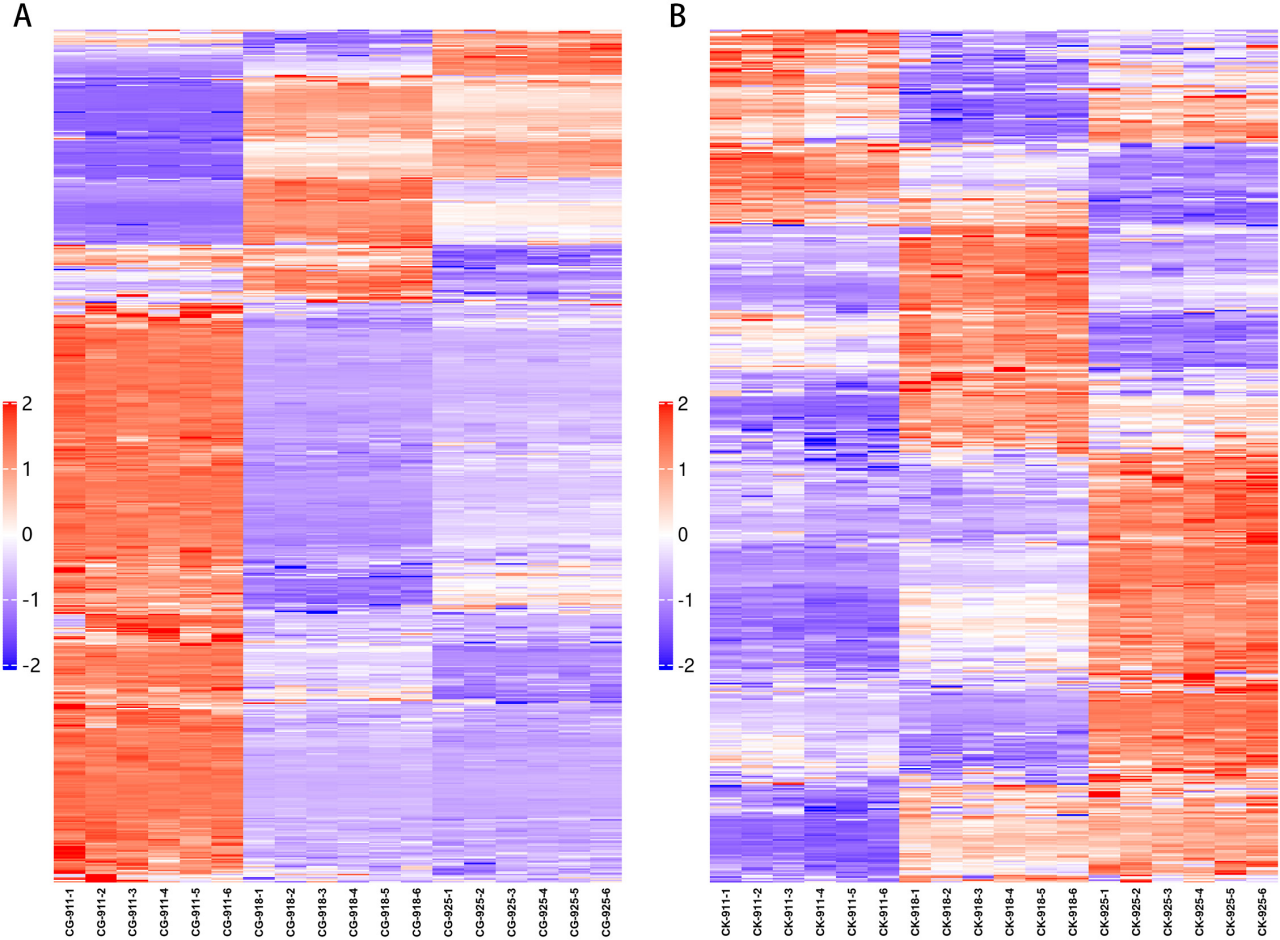
Heat map of fold-changes relative to treatment **(A)** and control **(B)** for detected metabolites in flower buds. In the figure, each row represents a differential metabolite (i.e., the metabolites with significant differences in expression are shown on the vertical axis), and each column represents a group of samples (i.e., the horizontal axis represents sample information). The color blocks at different positions represent the relative expression levels of the corresponding metabolites. Red indicates a relatively high expression level, while blue indicates a relatively low expression level. Metabolites with similar expression patterns are clustered together in the same cluster on the left side.

In the comparison of CG-911 vs CK-911, flavonoid biosynthesis was enriched and observed to be down-regulated without significant (**Figure 8A**). The main metabolic pathways enriched in CG-918 vs CK-918 and CG-925 vs CK-925 are illustrated in **Figures 8B, C**, 9 pathways were found as common to both comparisons. Notably, zeatin biosynthesis, ATP-Binding Cassette (ABC) transporters, the pentose phosphate pathway, the biosynthesis of phenylalanine, tyrosine, and tryptophan were activated in the CG groups. In contrast, the tricarboxylic acid (TCA) cycle, flavonoid biosynthesis, and pyruvate metabolism were down-regulated. Additionally, carbon metabolism and alanine, aspartate, and glutamate metabolism did not exhibit significant changes. Furthermore, cyanoamino acid metabolism was uniquely enriched and activated in the CG-925 vs CK-925 comparison. These findings suggest distinct metabolic pathway alterations associated with different developmental stages or treatments, highlighting the dynamic nature of metabolic regulation in response to experimental conditions.

Based on the enriched metabolic pathway analysis, a comprehensive screening of differential metabolites was conducted across the experimental groups. In the CK-911 vs CG-911 comparison, five significant metabolites were annotated. Among these, chlorogenic acid exhibited up-regulation, while four compounds showed down-regulation, including cianidanol, epicatechin, epigallocatechin, and gallocatechin. The CK-918 versus CG-918 comparison revealed more extensive metabolic alterations, with 6 down-regulated metabolites: L-malic acid, fumaric acid, sorbitol, cianidanol, chlorogenic acid, and epicatechin. Conversely, 12 metabolites demonstrated up-regulation: gluconic acid, gluconolactone, D-glucose, uridine, sucrose, choline, leucine, asparagine, shikimic acid, quinic acid, adenine and 5’-deoxy-5’-methylthioadenosine. The most substantial metabolic changes were observed in the CK-9.25 versus CG-925 group, where 18 differential metabolites were annotated. This group comprised 9 down regulated metabolites: gallocatechin, 4-hydroxy-3-methoxycinnamaldehyde, chlorogenic acid, cianidanol, D-glucose, epigallocatechin, fumaric acid, L-malic acid, and sorbitol. 9 upregulated metabolites, including 5’-deoxy-5’-methylthioadenosine, adenine, asparagine, gluconic acid, gluconolactone, phenylalanine, shikimic acid, uridine, and choline.

This comprehensive metabolic profiling provides valuable insights into the biochemical alterations associated with different experimental conditions, highlighting potential biomarkers and metabolic pathways for further investigation.

#### 3.2.4 Temporal changes in the metabolomic profile between treatment and control groups​

##### 3.2.4.1 Differential metabolites

An ANOVA analysis was performed to compare the three groups at various time points during the defoliation treatment and in the control group (**Figure 9**). Metabolites with a p-value < 0.05 were selected, revealing that the differential metabolites were predominantly concentrated in carbohydrates, fatty acids, flavonoids, amino acids, and prenol lipids. Notably, within the prenol lipids category, the treatment group exhibited a higher concentration of terpene glycosides, whereas the control group was characterized by a greater abundance of triterpenoids.

Specifically, when comparing the data from 9.18 with 9.11, a greater number of metabolites were altered in the treatment group compared to the control group. Among these, metabolites belonging to alcohols, polyols, and triterpenoids were up-regulated, while those associated with fatty acids and amino acids were down-regulated.

In the comparison between data from 9.18 and 9.25, the treatment group displayed a significantly higher number of down-regulated metabolites than the control group. The defoliated groups showed an increased presence of prenol lipids, whereas the control groups experienced a greater down-regulation of fatty acids. The number of up-regulated metabolites was relatively similar between the two groups, with both groups showing an accumulation of carbohydrates. Additionally, more prenol lipids were up-regulated in the control group.

##### 3.2.4.2 KEGG pathway enrichment

**Figure 10:**
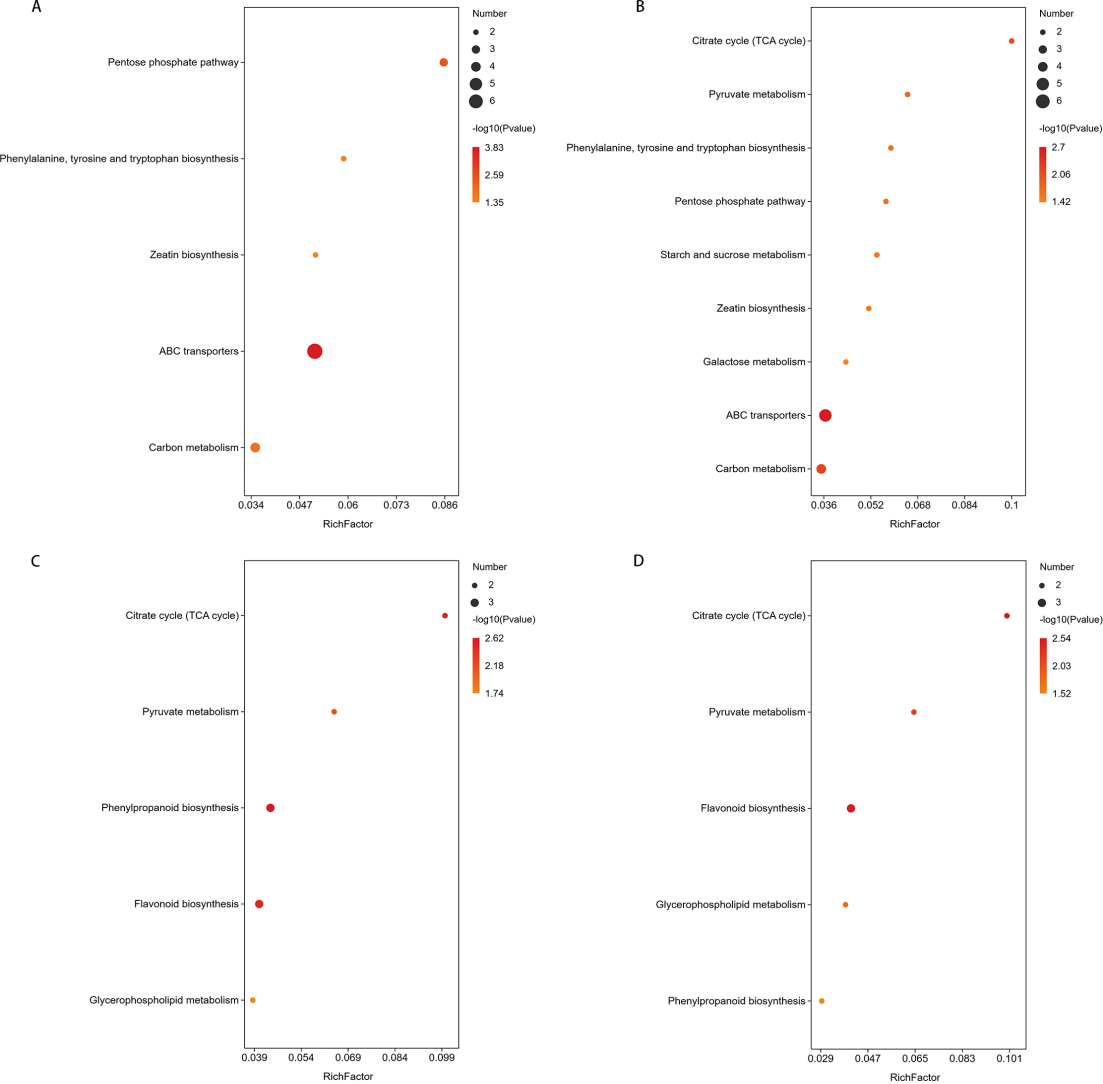
Metabolic pathway enrichment of differential metabolites. **(A-D)** represent the metabolic pathways enriched in the CG-918 vs CG-911, CG-925 vs CG-918, CK-918 vs CK-911,and CK-925 vs CK-918. The abscissa represents the Rich factor. The larger the graph, the more the number of different metabolites; the color indicates the significance of enrichment, that is, the p-value, the darker the red indicates that the pathway is more significantly enriched.

In the comparisons of CG-918 vs CG-911 and CG-925 vs CG-918 (**Figures 10A, B**), there are 5 and 9 pathways were enriched, respectively. Zeatin biosynthesis, ABC transporters, and phenylalanine, tyrosine, and tryptophan biosynthesis were activated in the first comparison (CG-918 vs CG-911) but depressed in the second (CG-925 vs CG-918). Carbon metabolism remained activated in both comparative groups. The pentose phosphate pathway was exclusively activated in CG-918 vs CG-911, whereas the TCA cycle and pyruvate metabolism were activated in CG-925 vs CG-918. Additionally, starch and sucrose metabolism and galactose metabolism were depressed in CG-925 compared to CG-918. The same KEGG pathways were enriched in both CK-918 vs CK-911 and CK-925 vs CK-918 (**Figures 10C, D**). Pyruvate metabolism and the TCA cycle remained consistently activated across both periods. Phenylpropanoid biosynthesis was only activated in the first period, while flavonoid biosynthesis was initially depressed but later activated in the second period.

#### 3.2.5 Important differential metabolites

##### 3.2.5.1 Effects of defoliation on carbohydrate metabolism and TCA cycle of pear flower buds

Carbohydrates serve as the primary energy storage substances and are integral components of cells, playing a crucial role in various plant life activities (Fettke and Fernie, 2015). The tricarboxylic acid (TCA) cycle, a central metabolic pathway in aerobic organisms, acts as a hub for carbohydrate, lipid, and amino acid metabolism. By facilitating oxidative metabolism in mitochondria, the TCA cycle generates ATP, and energy metabolism is a critical factor influencing the release of bud dormancy (Chen et al., 2020).

**Figure 11:**
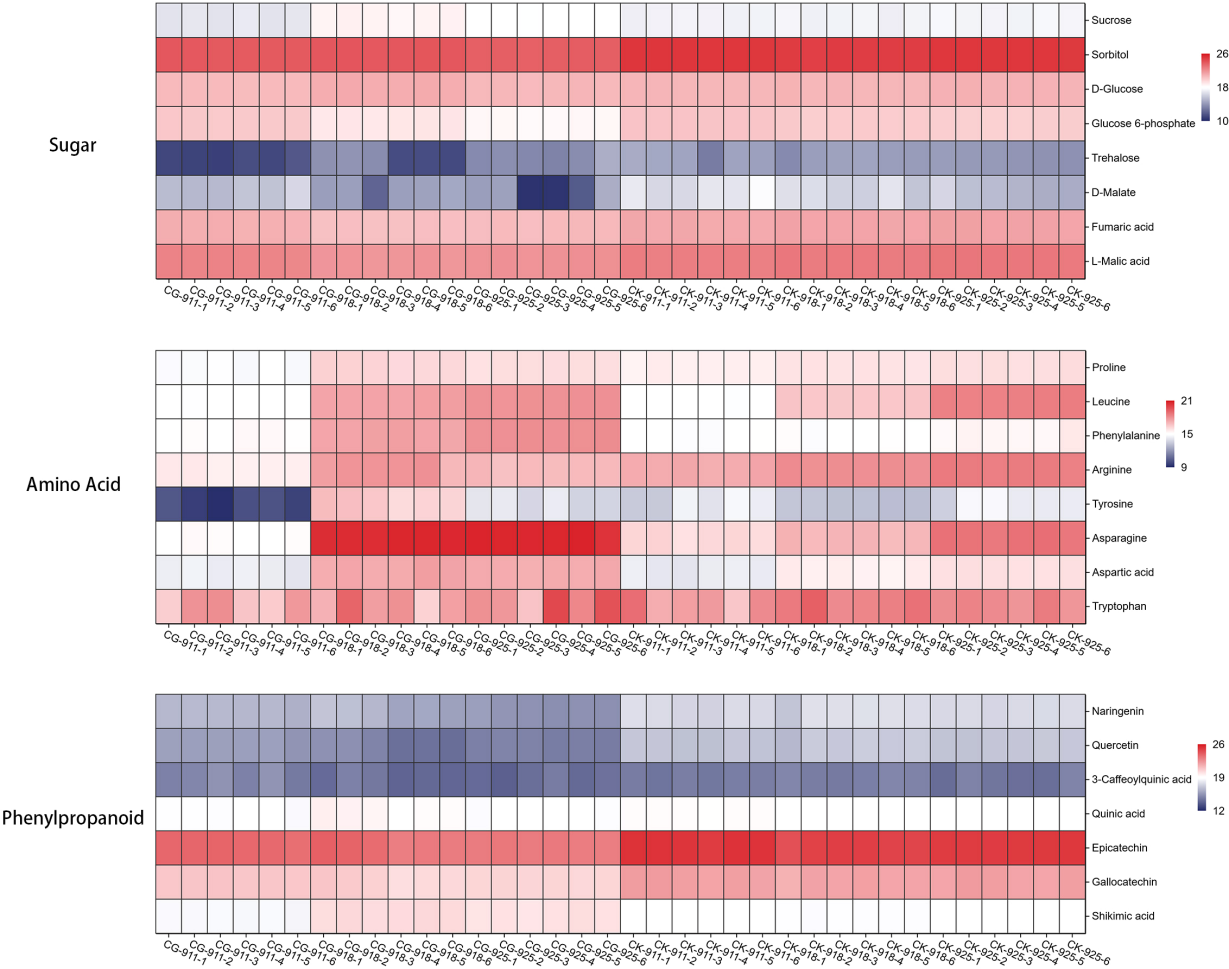
Heat map of fold-changes relative to sugar, amino acid and phenylpropanoid compounds changes in treatment and control groups. The data is normalized by log_2_ and used to draw a cluster heatmap, the numerical range indicated by the colors, with a gradient from dark blue to red, represents the values from low to high.

In the defoliation treatment group, most sugars exhibited a declining trend. Sucrose levels decreased significantly after 7 days of treatment but increased at 14 and 21 days. Notably, trehalose, sorbitol and glucose 6-phosphate were down-regulated over the 21-day period. Additionally, the levels of fumaric acid, D-malate, and L-malic acid, which are intermediates in the TCA cycle, were consistently lower in the defoliation treatment group compared to the control group (**Figure 11**).

##### 3.2.5.2 Effect of defoliation on amino acid metabolism of pear flower buds

Among the various metabolic networks, we focused on amino acid metabolism due to its critical role in plant physiology. Amino acids not only serve as the building blocks for protein synthesis but also act as precursors for the formation of diverse metabolites, playing essential roles in plant growth and stress responses (Binder, 2010). In our study, the amino acid content in the control group increased over time, whereas the defoliation treatment groups exhibited an opposite or more pronounced trend.

Specifically, proline showed gradual up-regulation in the control group but was significantly down-regulated after 7 days of treatment. However, it increased to levels similar to the control group at 14 and 21 days. Leucine was consistently and significantly up-regulated in the control group, with a more pronounced increase observed at 14 days defoliation treatment. Phenylalanine levels remained stable in the control group but were significantly up-regulated at 14 and 21 days after treatment. Arginine was continuously up-regulated in the control group but down-regulated in the treatment group. Asparagine, aspartic acid, and glutamine were steadily up-regulated in the control group, in the treatment group the up-regulation ratio was even higher. Tyrosine displayed a distinct pattern: in the control group, it was down-regulated at 14 days and then up-regulated at 21 days, whereas in the treatment group, it was down-regulated at 7 days and up-regulated at 14 days. In the control group, tryptophan levels initially increased and then decreased, whereas in the treatment group, they were continuously upregulated, reaching 1.5 times the level of the control group on day 21. These findings highlight the complex and dynamic responses of amino acid metabolism to defoliation treatment, underscoring its importance in plant adaptation to stress and growth regulation (**Figure 11**).

##### 3.2.5.3 Effects of defoliation on phenylpropane metabolism of pear flower buds

The phenylpropanoid metabolic pathway is a critical route in plants for synthesizing secondary metabolites, utilizing phenylalanine as a substrate through a series of enzymatic reactions. After 21 days of defoliation treatment, phenylalanine levels were significantly increased; however, among its downstream metabolites, only cinnamic acid showed a significant increase. In contrast, the contents of chlorogenic acid, ferulic acid, sinapic acid, caffeic acid, and coumarin were significantly reduced. Flavonoids, which are important branch products of the phenylpropanoid pathway, including naringenin, quercetin, epicatechin, and gallocatechin, were also significantly down-regulated (**Figure 11**).

Defoliation significantly promoted bud break, with rates of 6%, 47%, and 56% observed at 7, 14, and 21 days post-treatment, respectively, demonstrating statistically significant differences (p < 0.05) compared to the control (**Figure 1**). Metabolomic analysis revealed dynamic changes during bud release. Defoliation was found to significantly accelerate the morphological development of flower buds, as evidenced by anatomical comparisons with controls. Among the 1,533 annotated metabolites, lipids and lipid-like molecules (28.05%) and phenylpropanoids and polyketides (19.24%) were predominant. The number of differentially expressed metabolites increased markedly as bud break progressed, with prenol lipids, flavonoids, fatty acyls, and various organic oxygen compounds showing the most pronounced and sustained decreases. Key metabolites with the highest VIP scores included the down-regulated 9-octadecenamide, cianidanol, and octadecanamide, and the up-regulated 4a-carboxylic acid and choline. KEGG pathway analysis indicated that the defoliation treatment activated zeatin biosynthesis, ABC transporters, pentose phosphate pathway, and biosynthesis of phenylalanine, tyrosine, and tryptophan. In contrast, the TCA cycle, flavonoid biosynthesis, and pyruvate metabolism were down-regulated. Pathway-based screening identified metabolites with consistent trends: cianidanol, L-malic acid, fumaric acid, sorbitol, chlorogenic acid, and epigallocatechin were continuously down-regulated; while 5’-deoxy-5’-methylthioadenosine, adenine, asparagine, gluconic acid, gluconolactone, shikimic acid, uridine, and choline were persistently up-regulated. Sugars generally declined in the treatment group, though sucrose decreased initially and then increased. Key TCA cycle intermediates (fumaric acid, D-malate, L-malic acid) were consistently lower in the treatment group. Amino acid profiling showed distinct patterns: for instance, tryptophan was continuously increased in the treated buds, reaching 1.5 times the control level at day 21. Furthermore, despite a significant increase in phenylalanine after defoliation, most downstream metabolites in the phenylpropanoid pathway, such as chlorogenic acid, ferulic acid, and key flavonoids (naringenin, quercetin), were significantly down-regulated, suggesting a potential diversion of metabolic flux.

## 4 Discussion

Recently, the impact of reflorescence on pear production has gained increasing attention. However, the metabolic changes in buds induced by defoliation have rarely been explored. To elucidate the interference mechanism of defoliation on pear trees, we employed LC-MS/MS to analyze its effects on bud metabolism. By integrating the results from QC sample analysis, PCA, and PLS-DA, we confirmed the reliability of the metabolomics data, which can be confidently used for further analysis.

The bud burst percentages in flower buds gradually increased following defoliation treatment. Wei reported that even when 70% of leaves were removed, over 80% of buds were released after 40 days (Wei et al., 2022). In our study, bud burst rate reached 6%, 47% and 56% after 7, 14 and 21 days of defoliation, respectively (**Figure 1**). Through histological observations, it was evident that the development of flower buds in ‘Cuiguan’ pear was largely stagnant during the dormancy stage. Defoliation treatment significantly accelerated the process of flower bud morphological differentiation (**Figure 3**), particularly the flower primordium, which protruded on the 7^th^ day after leaf removal. This accelerated development process promoted the further expansion and maturation of flower organs, leading to the early germination of flower buds. These findings highlight the critical role of defoliation in disrupting dormancy and advancing flower bud development. Since the experiment was conducted in early autumn and the leaves of the control group had hardly dropped, we concluded that the flower buds were in a paradormancy state rather than endodormancy. The release of paradormancy has been closely associated with apical dominance, where lateral buds are inhibited by the apical bud, maintaining paradormancy (Cline and Deppong, 1999). In the Rose family, apical dominance typically regulates paradormancy, which is released after decapitation, leading to lateral bud outgrowth (Domagalska et al., 2011; Barbier et al., 2019). However, in this experiment, defoliation-induced paradormancy release occurred not only in lateral buds but also in top buds, suggesting that apical dominance alone cannot explain this phenomenon. Wei explain the phenomenon by IAA (indole-3-acetic acid) redistribution, pear leaves are a significant source of IAA, and defoliation alters IAA distribution along the stem, thereby inducing reflorescence (Wei et al., 2022). In our study, we focus on the overall changes in metabolites to speculate their relationship with flowering.

Based on our observations, the flowering process of reflorescence flower buds is similar to normal flower buds in spring. Therefore, we speculate that the changes in metabolic substances during the early stages of development are consistent with those of normal flower buds, which including the rise of sugars, amino acids, and protective secondary metabolites—underpins the successful organogenesis of normal floral buds. However, defoliation appears to adversely affect later developmental stages. This is compounded by issues such as a reduced number of flowers (typically 3–4 versus the normal 8-10), premature wilting (often associated with late-season low temperatures), and impaired fruit development. We hypothesize that these combined factors disrupt the normal flowering and fruiting processes.

A total of 1,533 metabolites exhibiting alterations were annotated, encompassing a diverse range of classes. In treatment groups, the number of differentially expressed metabolites increased markedly as bud break progressed, the predominant metabolites, lipids and phenylpropanoids, were both found to be down-regulated, which likely created a detrimental cycle where energy deficit integrity stalled development, while deficient antioxidant defenses led to premature wilting. There are 5 metabolites have the highest VIP values, down-regulation: 9-octadecenamide, cianidanol, and octadecanamide, up-regulation: 4a-carboxylic acid and choline. The down-regulation of 9-octadecenamide and octadecanamide, both fatty acid amides (Cheng et al., 2010), suggests disruptions in energy metabolism and lipid-derived signaling pathways critical for flower initiation. These compounds are implicated in stress responses and dormancy regulation, their reduction may reflect resource reallocation due to defoliation stress, leading to impaired floral organ development. Concurrently, the decline in cianidanol (a flavonoid antioxidant) indicates compromised oxidative defense (Liu et al., 2025), rendering petals vulnerable to reactive oxygen species (ROS) accumulation and accelerating premature wilting. Conversely, the up-regulation of choline—a phospholipid precursor—may regulate this process through its involvement in microRNA modulation (Niu et al., 2016) and gibberellin signaling (Chang and Sung, 2000). The physiological role of the accumulated 4a-carboxylic acid in floral bud development remains unclear, thus requiring further study. These findings highlight the dynamic metabolic changes associated with pear bud development and dormancy release.

These metabolites were enriched in various pathways, each playing distinct roles in the process of flowering. The defoliation treatment activated zeatin biosynthesis, ABC transporters, and the pentose phosphate pathway, which likely represents a stress response where the plant attempts to initiate cell repair and seek alternative energy sources in the face of carbon shortage (Körner, 2003). But pathways including TCA cycle, flavonoid biosynthesis, and pyruvate metabolism were inhibited. Studies have shown that the TCA cycle is enhanced during the dormancy release process in peach (Tang et al., 2019) and sweet cherry buds (Michailidis et al., 2018), playing a critical role in providing energy for bud germination. Horikoshi proposed that, at the end of endodormancy in pear buds, the TCA cycle is activated, facilitating carbohydrate metabolism and ATP generation to support flower bud germination (Horikoshi et al., 2018). In our study, the down-regulation of the TCA cycle and pyruvate metabolism may directly induce an energy crisis (Zhang and Liu, 2003), as evidenced by the reduction in key intermediates like L-malic acid and fumaric acid. The flowers might fail to provide sufficient energy to support subsequent development, which is likely associated with the early senescence commonly observed in reflorescence flowers after anthesis. Concurrently, the down-regulation of flavonoid and pyruvate metabolism led to a decline in total phenolics and flavonoids, an observation consistent with the metabolic reprogramming reported during flower development in *Rosa damascena* (Önder et al., 2022). This reduction likely reflects the consumption of these compounds in antioxidant defense, which would deplete the cellular antioxidant capacity, thereby increasing tissue susceptibility to oxidative stress and accelerating senescence.

Defoliation reduces the overall sugar levels in the plant, leading to a decline in sugar content within the buds (Kebrom et al., 2015). Buds are heterotrophic organs, meaning their germination and growth depend on both their internal nutrient reserves and their ability to absorb nutrients from surrounding tissues (Zapata et al., 2004; Greer et al., 2006). Sugars, as a critical category of nutrients, play a central role in various growth and developmental processes, including bud germination (Patrick et al., 2013; Barbier et al., 2015). The germination of buds relies heavily on the metabolism, transport, and signaling of sugars (Girault et al., 2010; Amelie et al., 2012; Chao et al., 2016). These processes involve a diverse array of sugar forms, such as starch, sucrose, monosaccharides, and sugar alcohols, among others (Chao et al., 2006). In this study, we observed that following defoliation, alongside the paradormancy release, the levels of sucrose, sorbitol, glucose, trehalose, and glucose-6-phosphate in flower buds changed.

Sucrose serves as a critical form of carbohydrate and energy transport in the phloem, facilitating long-distance movement between source and sink organs (Lemoine et al., 2013). During bud germination, the bud becomes a strong sink organ with a high demand for carbohydrates and energy, driving the transport of sucrose from source tissues to the bud, where it is metabolized and utilized (Bonhomme et al., 2010). In peaches, the presence of glucose and fructose has been linked to bud growth capacity (Maurel et al., 2004). Similarly, Hussain reported that low concentrations of glucose and fructose are associated with reduced bud break percentages in Japanese pear (Hussain et al., 2015). Moreover, in blueberry flower buds treated with hydrogen cyanamide, increased levels of fructose, glucose, and maltose provide both energy and signaling cues for germination (Wang et al., 2021). In our study, sucrose and D-glucose’s levels were lower in defoliation-treated buds after 7 days but increased significantly after 14 days and decreased to control level in 21 days; Glucose 6-phosphate level was continue lower than control group. With KEEG analysis we know that pentose phosphate pathway was activated after 14 days. We propose that, as a sink structure, the bud initially consumes a large amount of sucrose during the early stages of germination. Subsequently, it compensates for this demand by transporting from other parts of the plant. This dynamic regulation of sucrose levels highlights the importance of carbohydrate allocation and metabolism in supporting bud germination and growth under stress conditions.

In addition to sucrose, sorbitol is a significant form of sugar in *Rosaceae* plants, including pears and apples. It can be derived from the breakdown of starch and serves as a major sugar component in the xylem sap of pears (Nathalie et al., 2001; Ito et al., 2013). Sorbitol, a type of polyol, represents the reduced form of monosaccharides. Under abiotic stress conditions, polyols often replace sucrose to maintain cell turgor pressure and viability, accumulating in plants as a protective mechanism (Benjamin et al., 2007). (Williams and Raese, 1974) A study by Wei et al. on the impact of defoliation on reflorescence flowering showed that sorbitol levels in buds decreased following defoliation treatment (Wei et al., 2022). Our findings align with this observation, as sorbitol and trehalose’s content continued to decline after defoliation treatment. This reduction is likely due to the decomposition and utilization of sorbitol as an energy source during the bud germination process.

Amino acids serve as essential raw materials for protein synthesis and act as precursors for secondary metabolites, with their levels fluctuating in response to varying environmental conditions (Hildebrandt et al., 2015). Soluble proteins accumulate prior to flower bud release, which are then metabolized into amino acids serving as precursors for structural proteins and metabolic enzymes (Götz et al., 2017). When carbohydrate availability is limited, the energy demand can be met by the oxidation of amino acids (Galili et al., 2014).

Under stress conditions, arginine and proline play important roles. In the treatment group, the arginine level was continuously down-regulated, the proline level was lower than that in the control group on day 7 but returned to a level comparable to the control by day 21. We guess that during inflorescences flower differentiation, the stored proline and arginine are rapidly mobilized and consumed—proline is broken down to provide energy and carbon-nitrogen skeletons for flower bud development (Szabados and Savouré, 2010), while arginine is extensively used for polyamine synthesis to drive cell division and floral organ formation (Kawade et al., 2023). Furthermore, we observed an increase in 4-Guanidinobutyric acid levels. This may indicate a shift in arginine metabolism, whereby more arginine is converted into 4-Guanidinobutyric acid via arginine dehydrogenase, while proline production is inhibited (Morris, 2007).

Glutamine, aspartate, and asparagine serve as central regulatory hubs in carbon and nitrogen metabolism, interacting with a wide range of metabolic networks (Kirma et al., 2012; Xuemei et al., 2020). Glutathione, biosynthesized from glutamate, is crucial for the release of endodormancy in plants (Fuchigami and Nee, 1987). We observed that the level of glutathione decreased initially and subsequently increased after the treatment, whereas glutamine exhibited an opposite trend, with an initial increase followed by a decrease. At 21 days post-treatment, the glutathione concentration was 1.5 times higher than that in the control group. We hypothesize that the reconfiguration of the glutamine and glutamate metabolic flux following defoliation treatment is a core mechanism for the rapid enhancement of glutathione levels and improved stress resistance (Forman et al., 2008). Asparagine, with its relatively high C/N ratio (Xu et al., 2018), is a critical molecule for nitrogen storage and transport in plants, playing a vital role in regulating nitrogen assimilation and cycling (Xu et al., 2018). In blueberry flower buds treated with hydrogen cyanamide, aspartic acid levels decrease significantly, while threonine and homoserine, which are derived from aspartic acid, increase (Wang et al., 2021). Our findings, showing coordinated increases in aspartic acid (Asp) and asparagine (Asn). This is consistent with the established role of Asn as a key nitrogen transport molecule due to its high C/N ratio and stability (Xu et al., 2018). The upregulation likely represents a systemic response to reallocate nitrogen resources following the carbon and nitrogen crisis induced by defoliation (Gaufichon et al., 2013). The enhanced flux through Asp not only provides the precursor for Asn synthesis but also likely helps to maintain metabolic homeostasis, potentially by replenishing TCA cycle intermediates (Sweetlove et al., 2010).

Isoleucine, valine, leucine, and their derivatives, collectively known as branched-chain amino acids (BCAAs), play a vital role in regulating cellular osmotic balance, mitigating damage caused by reactive oxygen species (ROS), protecting membranes, and stabilizing proteins and enzymes (Xing and Last, 2017). Galili demonstrated that isoleucine acts as an electron donor, generating cellular energy and positively influencing bud development and germination (Galili et al., 2016). In blueberry flower buds, the levels of isoleucine and valine increased significantly, suggesting that the accumulation of BCAAs may promote bud germination (Wang et al., 2021). Conversely, in the study of Japanese pear ‘Housui’, leucine, isoleucine, and valine levels decreased during endodormancy under cold treatment, likely due to their catabolism into pyruvate to serve as an energy source (Horikoshi et al., 2018). In our study, although the leucine level in the defoliation group exhibited a continuous increasing trend between days 14 and 21, it ultimately showed no significant difference from the control group by day 21. The sustained accumulation of leucine represents an active stress adaptation response, potentially functioning in osmotic adjustment, antioxidant protection, and storing energy and precursors for germination (Xing and Last, 2017).

Phenylalanine, tyrosine, and tryptophan, as aromatic amino acids, Their synthesis was derived from the shikimate pathway, they are not only essential components of protein synthesis but also serve as precursors for a wide range of secondary metabolites. Phenylalanine serves as the primary substrate for the phenylpropanoid pathway, which produces numerous antioxidant metabolites crucial for stress responses, including lignin, flavonoids and anthocyanins (Fraser and Chapple, 2011). These secondary metabolites play a critical role in regulating plant growth, development, and defense responses. While tryptophan serves as a precursor for the synthesis of both auxin (IAA) and indole-type defense compounds, such as camalexin (Zhao, 2010). In blueberry flower buds, phenylalanine levels increased significantly, while tyrosine and tryptophan levels decreased (Wang et al., 2021). Similarly, our study revealed a significant increase in phenylalanine content following defoliation, accompanied by the upregulation of shikimic acid. This indicates that defoliation likely enhances phenylalanine biosynthesis through the shikimate pathway. The sustained accumulation of phenylalanine indicates a preferential allocation of resources into the phenylpropanoid pathway to enhance physical defense (e.g., lignification) and antioxidant capacity (Fraser and Chapple, 2011; Tohge et al., 2013). Tyrosine content was decreased after 7 days, but significantly elevated at 14 days, while tryptophan levels showed marked upregulation compared to the control by 21 days. The dynamic change in tyrosine—initially decreased followed by an increase—reflects an early metabolic realignment, whereas the marked upregulation of tryptophan at 21 days signals the activation of its dual functions: serving as a precursor for defense compounds and, more critically, as a substrate for auxin biosynthesis to promote flower bud germination (Zhao, 2010).

Secondary metabolites play a significant role in plant growth and development, yet their specific functions in the release of paradormancy in pear flower buds induced by defoliation treatment remain poorly understood. The phenylpropanoid biosynthesis pathway produces numerous phenolic compounds, which are known for their antioxidant and free radical scavenging properties (Savoi et al., 2016; Orrantia-Araujo et al., 2021). Oxidative stress is a critical process in dormancy release and bud germination (Muhammad et al., 2019). For instance, studies on raspberries have shown that an increase in germination rate correlates with the enhancement of the antioxidant system (Mazzitelli et al., 2007). Similarly, in blueberry flower buds, the accumulation of phenylpropanoids enhances the antioxidant capacity of the bud tissue (Wang et al., 2021). Horikoshi also observed increased levels of chlorogenic acid in pear buds during endodormancy under constant chilling treatment, which is essential for bud release (Horikoshi et al., 2018). In our study, despite the activation of the phenylalanine biosynthesis pathway and the consequent increase in phenylalanine content, the levels of most downstream metabolites—such as naringenin, quercetin, alpha-tocopherol, ferulic acid, sinapic acid, caffeic acid, coumarin, chlorogenic acid, epicatechin, and gallocatechin—were downregulated, whereas cinnamic acid was upregulated. This pattern contrasts with the regulation observed during normal flowering. The reduction in these secondary metabolites, which are crucial for antioxidant defense, may compromise the plant’s ability to manage oxidative stress during dormancy release. This lack of protection may explain why inflorescences flowers wither prematurely in defoliation-treated plants. These findings highlight the importance of phenylpropanoid metabolism in maintaining oxidative balance and supporting bud development. The down-regulation of key phenolic compounds under defoliation stress suggests a disruption in the antioxidant defense system, which may negatively impact bud viability and flowering. Furthermore, the observed increase in cinnamic acid content at both 14 and 21 days suggests the possible activation of the phenylalanine ammonia-lyase (PAL)-mediated pathway, which may promote lignin synthesis (Gao et al., 2020).

**Figure 12:**
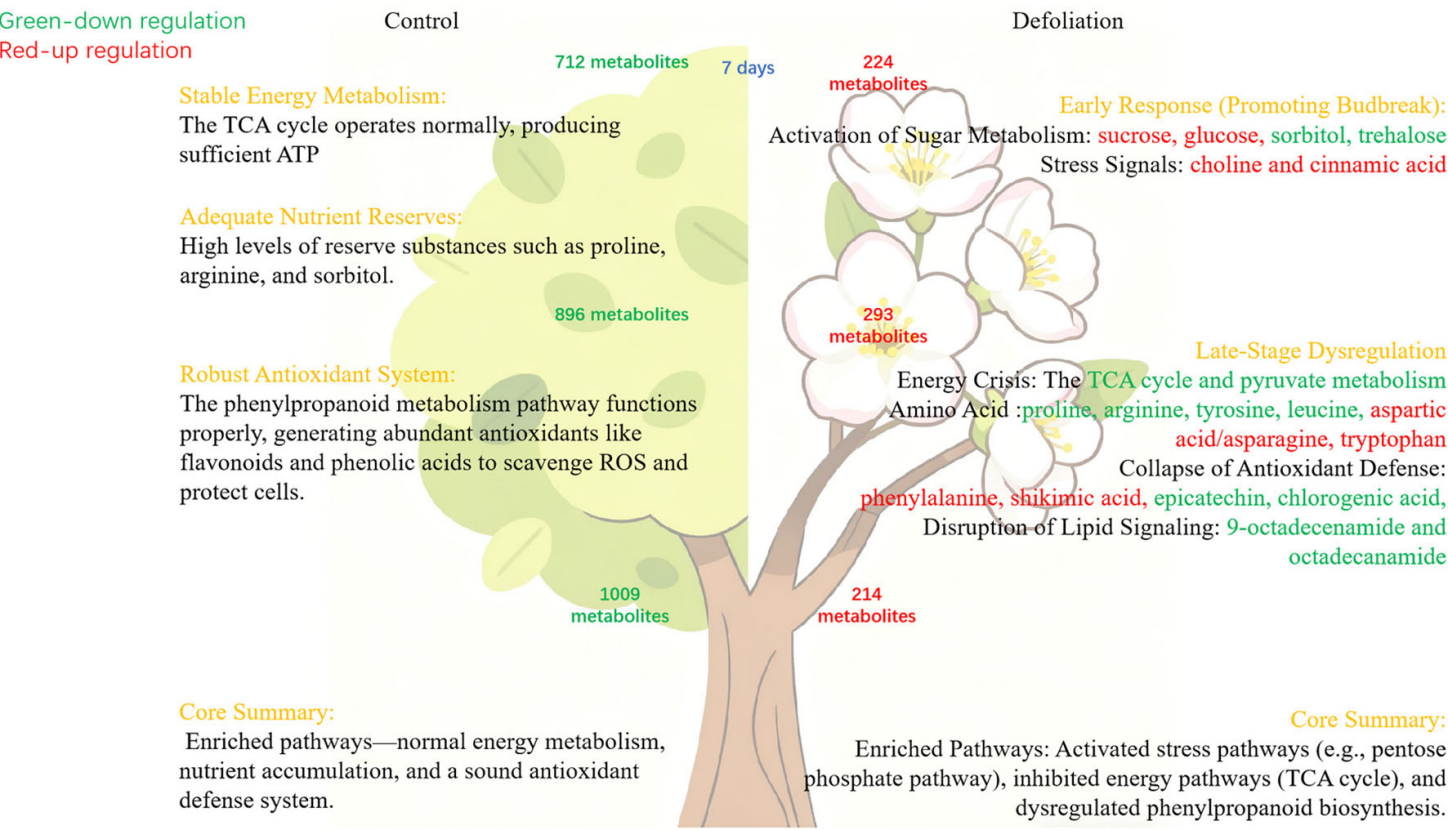
Metabolism changes in buds after defoliation.

This study provides a comprehensive elucidation of the physiological and metabolic mechanisms underlying defoliation-induced reflorescence in pear trees. It confirms that autumn defoliation treatment significantly promotes bud burst and accelerates the morphological differentiation of floral organs by releasing paradormancy in flower buds. Metabolomics analysis reveals that this process is accompanied by intense metabolic reprogramming. In terms of energy metabolism, the bud, acting as a strong sink, actively mobilizes sugars such as sucrose and sorbitol for energy in the early stages and activates the pentose phosphate pathway to cope with carbon shortage; however, the subsequent down-regulation of key intermediates in the TCA cycle and pyruvate metabolism indicates a severe energy crisis later on, which might be a crucial factor leading to premature wilting of the flowers. Regarding stress response, amino acid metabolism undergoes significant reprogramming: strategic reserves like proline and arginine are rapidly consumed to provide carbon and nitrogen skeletons, while the reconstitution of the glutamine-glutamate flux enhances glutathione levels to boost antioxidant capacity. Asparagine serves as key nitrogen transport/storage molecule due to high C/N ratio, upregulated post-defoliation for resource reallocation. BCAAs contribute to osmotic adjustment, ROS mitigation, and energy storage. Most critically, although the biosynthesis of phenylalanine is activated, the levels of most downstream phenylpropanoid compounds with antioxidant functions (e.g., flavonoids, phenolic acids) are generally down-regulated. This severely compromises the bud’s antioxidant defense system, rendering it unable to effectively scavenge reactive oxygen species (ROS), leading to oxidative damage and premature tissue senescence. In conclusion, while defoliation successfully induces bud burst, it concurrently causes disruptions in energy metabolism and a dysfunction in the antioxidant defense system. This internal metabolic imbalance ultimately results in the characteristic flaws of reflorescence: fewer flowers, impaired organ development, and premature senescence (**Figure 12**).

## 5 Conclusions

This study employs LC–MS/MS technology to systematically analyze metabolic changes during inflorescence flowering in pear trees following defoliation treatment, annotating 1,533 differentially expressed metabolites spanning nearly all major categories. Compared to control buds, inflorescence flower buds exhibited significant reductions in sugars, amino acids, and secondary metabolites. These alterations—rooted in the physiological process of inflorescence flowering—diverge from normal flowering metabolic pathways and may contribute to premature flower withering. The phenomenon disrupts the tree’s annual growth cycle, offering a valuable model for investigating bud differentiation, dormancy release, and flowering mechanisms. By comparing metabolite profiles between normal and inflorescence buds during differentiation, deeper insights into essential flowering mechanisms can be gained, supporting strategies for managing paradormancy and developing climate-resilient cultivars.

## Data Availability

The original contributions presented in the study are included in the article/**Supplementary Material**, further inquiries can be directed to the corresponding author/s.
