# Supplementary material for: Analysis of metabolites change from reflorescence buds of ‘Cuiguan’ pear (Pyrus pyrifolia) based on LC-MS/MS
Source: Front Plant Sci. 2025 Nov 21;16:1624304. doi: 10.3389/fpls.2025.1624304 (PMC12678315; doi:10.3389/fpls.2025.1624304)
Supplement: Supplementary file 1 [file DataSheet1.docx]

**supplementary material**


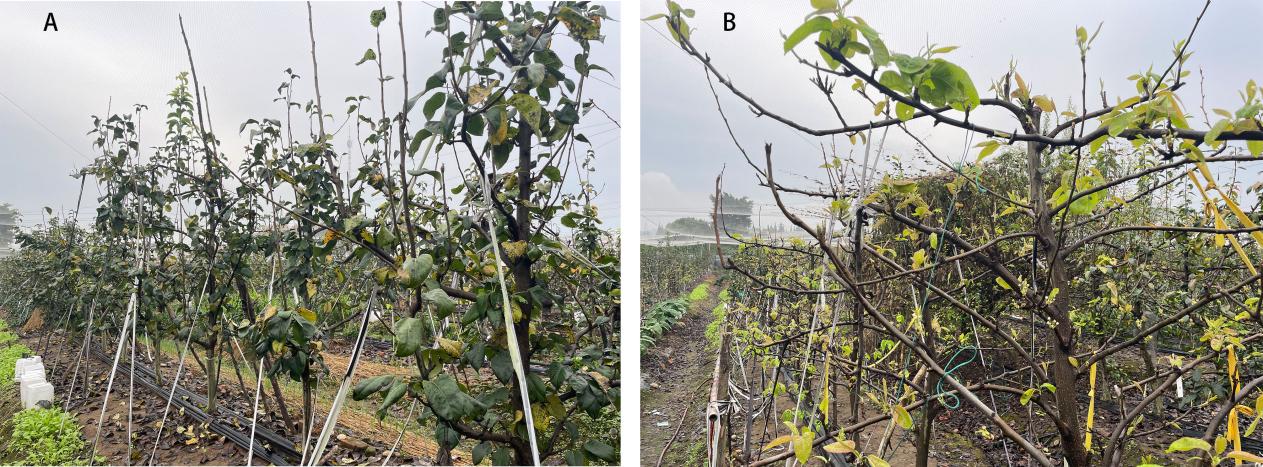


**Supplementary Figure 1.** Defoliation treatment on peer trees.A:The control group after 21 days, B: trees after defoliation treatment after 21 days


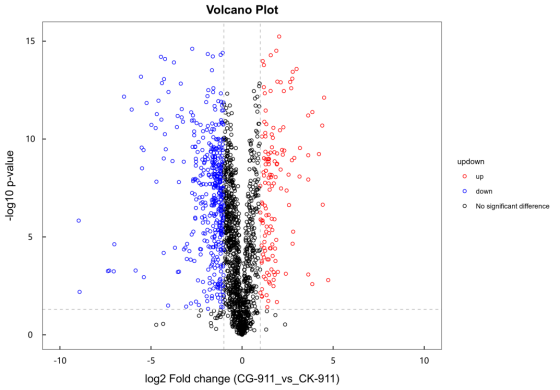

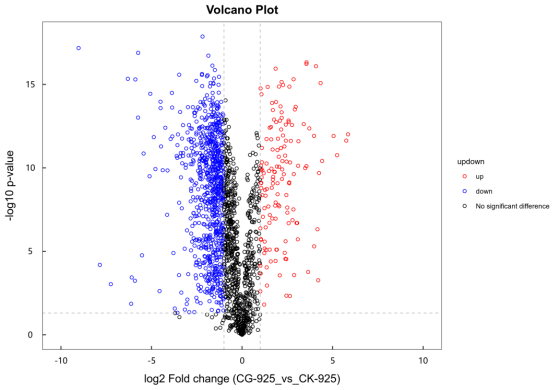

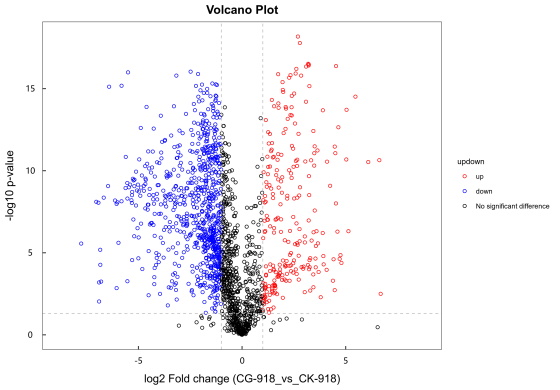


**Supplementary Figure 2.** the difference metabolite volcano diagram between the control group and experimental group. The blue circles represent significantly down-regulated metabolites, and the red circles represent significantly up-regulated metabolites

**Description of main characteristics of the assayed pear genotypes**

Genotype/Breed name：Pyrus pyrifolia ‘Cuiguan’

This variety is bred by the Institute of Horticulture, Zhejiang Academy of Agricultural Sciences, with 'Xingshui' as the female parent and the hybrid of 'Hangqing' and 'New Century' as the male parent. It exhibits prominent advantages including early maturity, high yield, and premium quality, paired with vigorous vegetative growth and strong environmental adaptability.

Officially recognized by the Crop Variety Approval Committee of Zhejiang Province in 1999, it has now been widely cultivated across regions such as Zhejiang, Chongqing, Sichuan, and Jiangxi. Notably, it is a leading cultivar and accounts for approximately 50% of the total production of early-maturing pear varieties in China, and its fruits have maintained high popularity in the market due to their excellent quality.

The fruit of this variety is round or oblong in shape, with a smooth yellowish-green peel dotted with a few rust spots. The average weight of a single fruit is 230g, while the maximum can reach up to 500g. Its flesh is snow-white, with a small core and minimal stone cells—boasting a fine, crisp texture, abundant juice, and a pleasant sweetness. The soluble solid content ranges from 11.5% to 13.5%, confirming its superior quality. In the Chengdu area, the fruit matures in late June.

The tree itself exhibits strong vigor, with a relatively upright growth habit. It is highly prone to flower bud formation, ensuring excellent and stable productivity. Additionally, it possesses strong resistance to adverse conditions and is well-suited for cultivation in diverse landscapes such as mountainous areas, plains, and tidal flats.
